# Effects of remimazolam and sevoflurane on postoperative delirium and early cognitive impairment in elderly patients after laparoscopic-assisted gastrointestinal surgery: a randomized clinical trial

**DOI:** 10.3389/fmed.2026.1680473

**Published:** 2026-03-20

**Authors:** Wei Ren, Kai Zheng, Jian Song, Li Zhang, Shenqiang Gao

**Affiliations:** 1 Department of Anesthesiology, The Affiliated Taian City Central Hospital of Qingdao University, Taian, Shandong, China; 2 Department of Painology, The Affiliated Taian City Central Hospital of Qingdao University, Taian, Shandong, China

**Keywords:** early cognitive impairment, elderly patients, postoperative delirium, remimazolam, sevoflurane

## Abstract

**Background:**

Postoperative delirium (POD) is a prevalent complication after surgery and anesthesia in elderly patients. Remimazolam and sevoflurane are two commonly used general anesthetics. This study aimed to evaluate the effects of remimazolam and sevoflurane on POD and early postoperative cognitive function in elderly patients.

**Methods:**

A total of 90 patients scheduled for laparoscopic-assisted gastrointestinal surgery under general anesthesia were recruited, and randomly assigned into three groups (n = 30 for each group): remimazolam (R), sevoflurane (S), and remimazolam combined with sevoflurane (RS) groups. After surgery, the incidence of POD and early postoperative cognitive impairment, postoperative pain score, recovery quality score, intraoperative drug dosage, and the incidence of intraoperative adverse reactions were assessed.

**Results:**

No significant differences were observed in the POD incidence (*p* = 0.654), occurrence time (*p* = 0.985), duration (*p* = 0.355), and classification (*p* = 1.000) among the R, S, and RS groups. The incidence of delayed recovery of post-operative cognitive function also did not differ significantly among the three groups (*p* = 0.616). Additionally, the incidence of intraoperative hypotension was lower, and the use of vasoactive drugs was reduced in the R group (*p* < 0.05). When combined with sevoflurane, the induction and maintenance doses of remimazolam were reduced, and the time to loss of consciousness was shortened (*p* < 0.05)..

**Conclusion:**

Remimazolam was not associated with an increased incidence of POD or delayed recovery of early cognitive function in elderly patients. Moreover, it had a less pronounced impact on circulation compared to sevoflurane.

**Clinical trial registration:**

https://www.chictr.org.cn/showproj.html?proj=174731, identifier ChiCRT2200064984.

## Background

Postoperative delirium (POD) is an acute cognitive dysfunction characterized by impairments in memory and consciousness (1). Generally, delirium is prevalent among the elderly, particularly in those with pre-existing neurocognitive disorders or following insults like infection or trauma (2). It is reported that the overall incidence rate of POD is approximately 23%, varying depending on the type of surgery the patient undergoes (3). In high-risk surgeries, the prevalence can reach as high as 50%. POD can prolong the hospitalization time of patients, and increase their one-month mortality (4). Furthermore, POD not only has detrimental effects on patients but also increases the burden on clinical doctors, as it increases hospitalization time, medical costs, nursing burden, risk of complications, and mortality, becoming an increasingly serious public health problem all around the world (5, 6). The etiology of POD is multifactorial, encompassing triggering and inducing factors (7). Evidence indicates that performing the same surgery under different anesthetics may result in different rates of postoperative cognitive impairment (8–10).

Sevoflurane, an inhaled anesthetic used to induce and maintain general anesthesia, is considered a safe clinical reagent and is commonly employed in both adult and pediatric anesthesia (11). Characterized by a low blood gas coefficient, rapid onset, and quick recovery, it has been a staple in the operating room. However, recent research has indicated that sevoflurane exerts neurotoxic effects on the central nervous system, manifested as cognitive impairment and abnormal behavior (12, 13). A prior study by Cao et al. (14) demonstrated that, among elderly patients undergoing major cancer surgery, the incidence of POD was higher among those receiving sevoflurane anesthesia (12.4%) than among those receiving propofol anesthesia (8.4%). Another randomized clinical trial reported that the incidence of POD following sevoflurane anesthesia in elderly patients undergoing total hip/knee replacements was 23.3% (24/103) (15). Furthermore, sevoflurane inhalation anesthesia may lead to cognitive dysfunction in a dose-dependent manner (16). Consequently, there is a pressing need to actively seek alternative anesthetics or anesthesia regimens for surgical procedures.

Remimazolam, a novel ultra-short-acting benzodiazepine drug, has recently been approved for programmed sedation and general anesthesia (17). Its metabolism predominantly depends on tissue esterase induction, is independent of liver and kidney function, and its metabolites are inactive (18). Clinical trials have shown that remimazolam can provide rapid-onset sedation and a quick recovery, while minimizing respiratory depression and blood pressure disturbances (19, 20). As such, it appears to be an ideal intravenous anesthetic for elderly surgical patients. However, previous studies have suggested that benzodiazepines increase the risk of POD development (21, 22), and guidelines in the United States and Europe recommend that elderly surgical patients minimize the use of benzodiazepines (23, 24). Emerging studies have investigated the efficacy and safety of remimazolam in elderly patients (aged ≥60 years) undergoing various surgeries, including cardiovascular and orthopedic procedures, and demonstrated that remimazolam does not increase the incidence of POD and is associated with favorable hemodynamic stability (25, 26). A recent randomized controlled trial has also explored the combination of remimazolam and sevoflurane for abdominal surgery, revealing a potential synergistic effect in reducing anesthetic dosage (27). As a new benzodiazepine drug, remimazolam has a significantly lower incidence of POD than propofol, and shows no significant correlation with POD compared with other anesthetics (28, 29). A recent investigation reported that in children who underwent tonsillectomy and adenoidectomy, administering remimazolam at the end of the operation significantly decreased the likelihood of POD after sevoflurane anesthesia, compared to 0.9% saline (30). Nevertheless, the effects of remimazolam on POD incidence in elderly patients undergoing laparoscopic-assisted gastrointestinal surgery, a specific subgroup with high comorbidity and unique physiological characteristics, as well as its direct comparison with sevoflurane inhalation anesthesia, and the clinical outcomes of their combination in this population, remain to be systematically elucidated.

Although the independent effects of remimazolam and sevoflurane on postoperative neurocognitive outcomes have been partially explored (31–34), the clinical significance of their combined use, which is increasingly common in clinical practice, has not been comprehensively evaluated. In the routine anesthesia management of elderly patients undergoing laparoscopic-assisted gastrointestinal surgery, a balanced strategy is often required to reconcile the need for stable hemodynamics (a potential advantage of remimazolam) and rapid anesthetic induction/maintenance (a key feature of sevoflurane) (35). Clinicians frequently combine low-dose intravenous anesthetics with inhaled anesthetics to optimize induction speed while mitigating the adverse effects of high-dose single-agent use, such as excessive hypotension from high-dose remimazolam or prolonged neurocognitive disturbances from high-dose S. From a pharmacodynamic perspective, preliminary preclinical and observational studies have suggested a potential synergistic interaction between benzodiazepines and inhaled anesthetics. The combination may reduce the minimum alveolar concentration (MAC) of sevoflurane and the required dose of remimazolam, thereby decreasing the risk of drug accumulation-related side effects (36, 37). However, it remains unknown whether this synergistic effect translates into improved clinical outcomes, specifically, whether the combination of remimazolam and sevoflurane can maintain the favorable hemodynamic profile of remimazolam while retaining the rapid induction advantage of sevoflurane, without increasing the incidence of POD or early cognitive impairment. Furthermore, elderly patients undergoing laparoscopic gastrointestinal surgery often present with comorbidities (e.g., hypertension, cardiovascular disease) and reduced physiological reserve, making them highly sensitive to anesthetic-related hemodynamic fluctuations and neurotoxicity (38, 39). The combination of remimazolam and sevoflurane was thus designed to test a clinically relevant hypothesis: whether combining these two drugs can achieve a “balance” of anesthetic efficacy and safety (i.e., reducing individual drug doses, shortening induction time, and maintaining hemodynamic stability) without compromising neurocognitive outcomes compared to single-agent use. This exploration is critical for guiding evidence-based selection of anesthesia regimens in this high-risk patient population.

Therefore, we designed a randomized controlled trial to assess the impacts of intraoperative remimazolam and sevoflurane application on POD and early postoperative cognitive function in elderly patients undergoing laparoscopic-assisted gastrointestinal surgery.

## Materials and methods

### Ethics approval

This study was a single-center, double-blind, prospective, randomized controlled clinical trial conducted at Taian City Central Hospital in Taian, China. The research protocol was approved by the Ethics Committee of Tai’an Central Hospital (approval no. 2022-6-27) and registered in the Chinese Clinical Trial Registry with the registration number of ChiCRT2200064984 (https://www.chictr.org.cn/showproj.html?proj=174731). All participants provided written informed consent before study enrollment.

### Patient recruitment and grouping

From August 2023 to February 2024, 90 patients scheduled for laparoscopic-assisted gastrointestinal surgery under general anesthesia were recruited at Tai’an Central Hospital. The inclusion criteria are as follows: age ≥ 65 years, American Society of Anesthesiologists (ASA) grade I to III, and the ability to cooperate with neuropsychological tests. The exclusion criteria are as follows: (1) Patients with severe cardiovascular, respiratory, liver, kidney or central nervous system diseases; (2) Patients with cognitive dysfunction or delirium before surgery; (3) Patients with a history of dementia or mental illness; (4) Patients who currently used sedatives, antidepressants, or corticosteroids; (5) Patients with alcoholism and drug dependence; (6) Patients with difficult follow-up or poor compliance (such as aphasia, blind people, etc.); and (7) Patients with contraindications related to experimental drugs. Additionally, the cognitive function of all patients was assessed using the Mini-Mental State Examination (MMSE) for adults on the day before surgery. Patients with cognitive impairment, stratified by educational level according to the following criteria, were excluded from the trial: an MMSE score of < 17 for illiterate patients, < 20 for patients with primary school education, and < 24 for patients with secondary school education or higher (including technical secondary school).

Before the operation, a member of our research team visited the patients to determine their eligibility for the trial based on the inclusion and exclusion criteria. Written informed consent was obtained from all patients prior to the study. Subsequently, all participants were randomly assigned to one of three groups using randomization numbers: remimazolam (R), sevoflurane (S), and remimazolam combined with sevoflurane (RS) groups. A non-research team member performed the randomization, and the random numbers were sealed in sequentially numbered envelopes and stored at the study site. On the day of surgery, enrolled patients entered the operating room, and the corresponding experiments were carried out according to the random number in the sealed envelope. Additionally, both the patients and post-operative data collectors were blinded to the grouping.

### Anesthesia and surgery

All patients refrained from using preoperative drugs and followed routine dietary restrictions. Upon entering the operating room, pulse oximetry (SpO2), electrocardiography (ECG), and non-invasive blood pressure (NIBP) were monitored. The left upper-limb vein was cannulated, and left brachial artery puncture was performed under local anesthesia. Anesthesia induction was as follows: after 0.5 μg/kg of sufentanil, patients in the R group were induced with remimazolam at 6 mg/kg/h until loss of consciousness, after which anesthesia was maintained at a dose of 0.5–2 mg/kg/h. In the S group, patients received 0.5 μg/kg of sufentanil, followed by 8% sevoflurane (S) (oxygen flow 8 L/min), and anesthesia was maintained with 1–2% sevoflurane after loss of consciousness. For the RS group, after 0.5 μg/kg of sufentanil, patients were induced with 6 mg/kg/h remimazolam while inhaling 8% sevoflurane. After loss of consciousness, anesthesia was maintained with 0.5–2 mg/kg/h remimazolam combined with 0.4–2% sevoflurane. The dosages were within a range and adjusted as needed based on BIS values and changes in the patient’s vital signs to ensure perioperative safety. Therefore, the simultaneous use of the two anesthetics in the RS group did not result in excessive anesthesia.

After loss of consciousness, patients in all three groups were given 0.6 mg/kg of rocuronium bromide, and endotracheal intubation was performed 90 s later. The inhaled oxygen concentration was set at 50%, the tidal volume at 8 mL/kg, the respiratory ratio at 1: 2, and the respiratory rate was adjusted to maintain an end-expiratory carbon dioxide partial pressure of 35–45 mmHg. After intubation, 80 mL of 0.25% ropivacaine hydrochloride was used for ultrasound-guided rectus abdominis sheath and transverse abdominis plane block. During anesthesia maintenance, remifentanil was continuously transfused at 0.10–0.20 μg/kg/min, and 0.15 mg/kg rocuronium bromide was intermittently administered to maintain muscle relaxation, while remimazolam and sevoflurane were adjusted to keep Bispectral Index (BIS) values of 40–60. Half an hour before the end of the surgery, 0.15 μg/kg of sufentanil was injected intravenously, and glucocorticoids, non-steroidal analgesics, and dexmedetomidine were not used during the surgery. The anesthesiologist selected the appropriate vasoactive drugs (ephedrine, norepinephrine, and or phenylephrine) according to the condition of patients, recorded the consumption of vasoactive drugs, and ultimately converted them into norepinephrine equivalents (1 mg ephedrine was equivalent to 1 μg norepinephrine, and 100 μg phenylephrine was equivalent to 7.6 μg norepinephrine). Post-surgery, the patient received a sufentanil-controlled analgesia pump for 48 h and was transferred to the resuscitation room for extubation. All anesthesiologists involved in this study held the professional title of attending physician or above, and all received unified standardized training before the trial. During the study, the anesthesiologists strictly adhered to the predefined protocol and documented all relevant data while ensuring the safety of all patients throughout the process. Furthermore, postoperative follow-up and outcome assessment were undertaken by other independent researchers from the research team, who were not involved in patient screening, randomization, or any aspect of anesthetic management for the trial.

### Measurement of observational indices

The observational indices comprise primary indicators and secondary indicators. Among them, the primary indicators included the incidence of POD using the 3-min Diagnostic Interview for Confusion Assessment Method (3D-CAM). Briefly, each participant was assessed twice a day (8:00–10:00, and 18:00–20:00) on the 1st, 2nd, and 3rd postoperative days, respectively, by a study member who had received training before the study and had not participated in patient clinical care (detailed in the Supplementary materials 1. Measurement of observational leading indicators). Based on the Richmond Sedation Scale (RASS) (detailed in the Supplementary materials 1. Measurement of observational leading indicators), POD patients were further classified into three subtypes according to the level of consciousness before delirium assessment: high active delirium with consistently positive RASS (+1 to +4), low active delirium with consistently neutral or negative RASS (−3 to 0), and mixed delirium with the occurrence of simultaneous episodes of high and low activity delirium during the observation period; as well as the onset time and duration of delirium were recorded (40).

The secondary indicator included the incidence of early postoperative cognitive impairment in adults, as measured by MMSE (detailed in Supplementary materials 2. Measurement of the incidence of delayed recovery of postoperative cognitive function using MMSE). In brief, all participants were tested and recorded on the first day before surgery, and on the 1st, 2nd, and 3rd postoperative days, respectively, to evaluate whether there was postoperative cognitive impairment (MMSE score < 17 for illiteracy, < 20 for primary school, < 24 for secondary school including vocational school, or a score 2 points lower than that before surgery considered as cognitive impairment). If the patient develops POD, the evaluation will be postponed until the recovery from POD. In addition to POD evaluation, the Pain Numeric Rating Scale (NRS) was used to assess the degree of postoperative pain; the QoR-15 scale was employed to evaluate the postoperative recovery of patients; as well as the dosage of remimazolam used during induction until the end of intubation, the total amounts of remimazolam, remifentanil, and sufentanil used during surgery were assessed. Furthermore, the incidence of intraoperative adverse reactions, including intraoperative hypertension, hypotension, tachycardia, bradycardia, arrhythmia, injection pain, nausea, vomiting, and dizziness, was observed (detailed in the Supplementary materials and Supplementary methods 3. Adverse event definitions). The need for remedial sedatives, intraoperative movement, intraoperative awareness, and agitation during the recovery period was also evaluated.

### Statistical analysis

Statistical analysis was conducted using SPSS 25.0 software. Metric data were expressed as mean ± standard deviation (SD) or median (interquartile range, IQR), and inter-group comparisons were performed using one-way analysis of variance (ANOVA) or Kruskal-Wallis rank sum test. For the comparisons of the enumeration data, the Chi-square test or Fisher’exact probability method was performed. To avoid the risk of false positives associated with multiple comparisons, the Bonferroni correction was applied to post-hoc analyses following one-way ANOVA or the Kruskal-Wallis rank-sum test for multiple independent samples, and all reported *p*-values are corrected for multiple comparisons. *p* < 0.05 was considered statistically significant. As for the calculation of sample size, the incidence of POD in elderly patients undergoing gastrointestinal surgery under intravenous anesthesia and inhalation anesthesia was, respectively, 6.9 and 26.7% according to the previous literatures (41, 42), and 30 cases in each group of 90 samples were sufficient to achieve the double-tail *α* = 0.05, 80% test efficacy, and 10% dropout rate.

## Results

### Clinical characteristics of all participants

**Figure 1:**
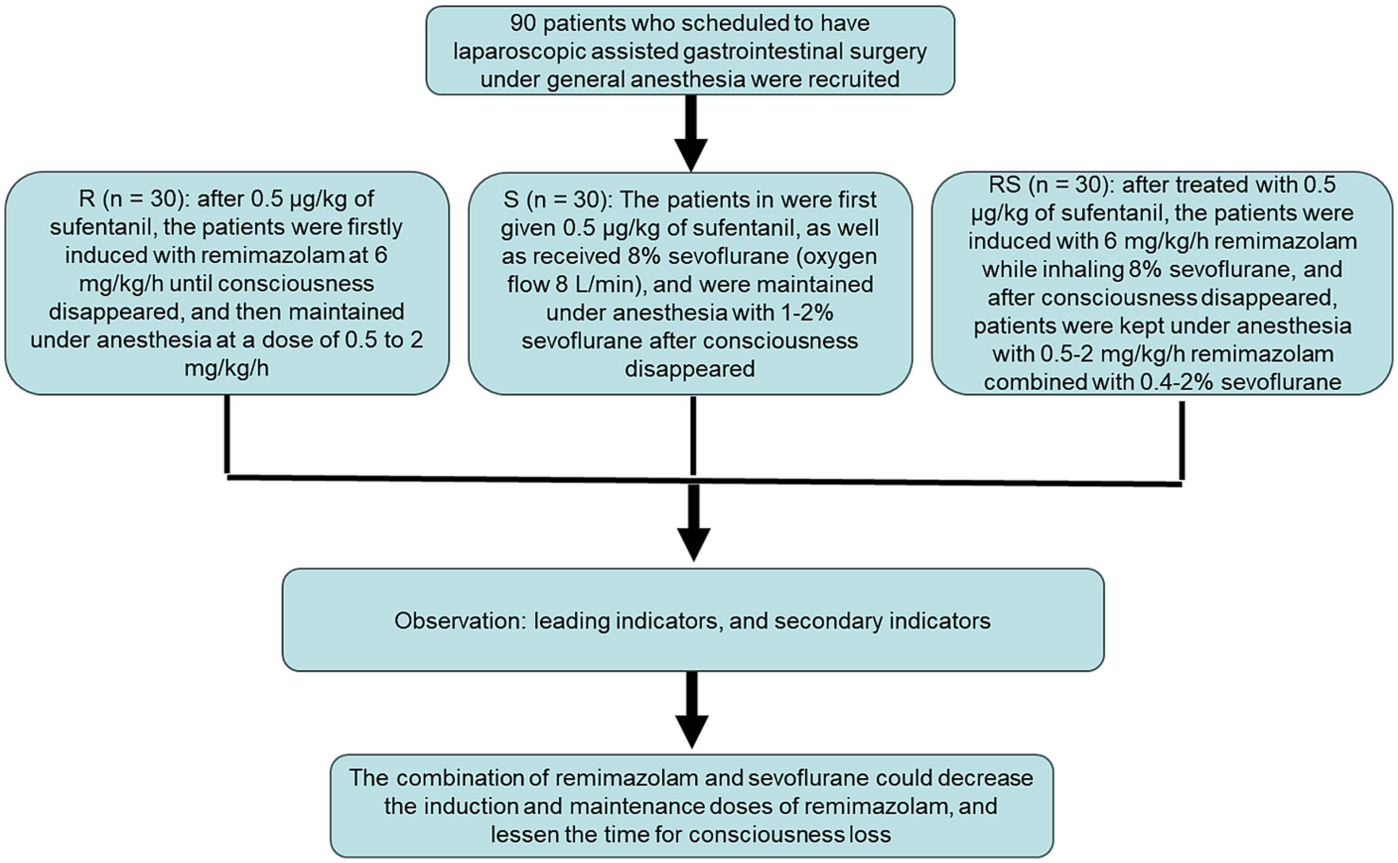
The flow diagram of this study.

The study flow diagram is presented in Figure 1. In this study, a total of 90 participants were recruited and randomly allocated into three groups (R, S, and RS groups) with 30 patients per group. After comparing the basic clinical information of the patients in these three groups, no significant differences were found in gender, age, body mass index (BMI), American Society of Anesthesiologists (ASA) classification, asthenia stage, level of education, drinking, smoking, preoperative complications, and operative site among the R, S and RS groups (*p* > 0.05, Table 1). The values of age-adjusted Charson comorbidity index in the R, S and RS groups were, respectively, 3.5 ± 1.0, 3.6 ± 0.9, and 3.5 ± 1.0; as well as the values of MMSE one day before surgery were, respectively, 27.5 ± 2.4, 27.6 ± 1.9, and 28.0 ± 2.1, which showed no significant differences among the three groups (*p* > 0.05, Table 1). Additionally, no significant differences were observed in operation duration, total liquid intake, amount of bleeding, urine volume, and transfusion ratio among the R, S, and RS groups (*p* > 0.05, Table 1). However, the dosages of norepinephrine in the R, S, and RS groups were evidently different (*p* < 0.05), with the highest in the S group, followed by the RS and R groups (Table 1).

**Table 1:** Clinical characteristics of all participants in the three groups.

Group	R (*n* = 30)	S (*n* = 30)	RS (*n* = 30)	*χ*^2^/F/H	*P*
Gender (male/female)	20/10	21/9	22/8	0.317	0.853
Age (years old)	68.0 ± 2.4	68.9 ± 3.4	67.8 ± 1.8	1.602	0.207
BMI (kg/m^2^)	23.3 ± 3.0	22.3 ± 3.1	23.6 ± 3.2	1.591	0.210
ASA (II/III)	26/4	23/7	27/3	2.050	0.439
Frailty stage	*n* (%)			1.170	0.557
Non-frailty	14 (46.7)	11 (36.7)	15 (50)		
Pre-frailty	16 (53.3)	19 (63.3)	15 (50)		
Level of education	*n* (%)			1.089	0.912
Illiteracy	4 (13.3%)	4 (13.3%)	4 (13.3%)		
Primary school	8 (26.7)	5 (16.7)	7 (23.3%)		
Secondary school and above	18 (60.0%)	21 (70.0%)	19 (63.3%)		
Drinking [*n* (%)]	12 (40.0%)	11 (36.7%)	14 (46.7%)	0.643	0.800
Smoking [*n* (%)]	6 (20.0%)	8 (26.7%)	9 (30.0%)	0.818	0.664
Preoperative complications	*n* (%)				
Hypertension	14 (46.7%)	16 (53.3%)	13 (43.3%)	0.623	0.732
Diabetes	2 (6.7%)	4 (13.3%)	5 (16.7%)	1.482	0.611
CHD	5 (16.7%)	7 (23.3%)	4 (13.3%)	1.064	0.587
CVD	4 (13.3%)	5 (16.7%)	8 (26.7%)	1.886	0.390
aCCI	3.5 ± 1.0	3.6 ± 0.9	3.5 ± 1.0	0.084	0.919
MMSE 1 day before surgery	27.5 ± 2.4	27.6 ± 1.9	28.0 ± 2.1	0.352	0.704
Operative site	*n* (%)			2.219	0.709
Stomach	20 (66.7%)	23 (76.7)	18 (60.0%)		
Rectum	4 (13.3%)	3 (10.0%)	6 (20.0%)		
Colon	6 (20.0%)	4 (13.3%)	6 (20.0%)		
Operation duration (min)	181.3 ± 50.3	193.6 ± 54.0	183.1 ± 39.8	0.563	0.572
Total liquid intake (mL)	2500.0 (1000.0)	2500.0 (500.0)	2500.0 (125.0)	1.474	0.412
Amount of bleeding (mL)	100.0 (137.5)	100.0 (137.5)	100.0 (50.0)	0.270	0.874
Urine volume (mL)	450.0 (375.0)	325.0 (200.0)	400.0 (375.0)	0.070	0.965
Transfusion ratio [*n* (%)]	2 (6.7%)	3 (10.0%)	1 (3.3%)	1.102	0.868
Length of stay	15.1 ± 6.1	17.0 ± 5.1	14.4 ± 4.3	2.116	0.127
Dosage of norepinephrine (μg)	5.0 (8.0)	25.0 (30.5)	10.0 (31.0)	10.301	0.006

BMI, body mass index; ASA: American Society of Anesthesiologists; CHD, coronary heart disease; CVD, cerebrovascular disease; MMSE, mini-mental state examination; aCCI, Age-adjusted Charson comorbidity index. R: After 0.5 μg/kg of sufentanil, the patients were first induced with remimazolam at 6 mg/kg/h until consciousness disappeared, and then maintained under anesthesia at a dose of 0.5 to 2 mg/kg/h. S: The patients were first given 0.5 μg/kg of sufentanil, as well as received 8% sevoflurane (oxygen flow 8 L/min), and were maintained under anesthesia with 1–2% sevoflurane after consciousness disappeared. RS: After being treated with 0.5 μg/kg of sufentanil, the patients were induced with 6 mg/kg/h remimazolam while inhaling 8% sevoflurane, and after consciousness disappeared, the patients were kept under anesthesia with 0.5–2 mg/kg/h remimazolam combined with 0.4–2% sevoflurane.

### Incidence and severity of POD in the three groups

Among all participants in this study, the overall incidence of POD was 14.4% (13/90), of which four patients (13.3%) in the R group, six patients (20.0%) in the S group, and three patients (10.0%) in the RS group, which showed no statistical difference in the incidence of POD among the three groups (*p* = 0.654 > 0.05, Table 2). Furthermore, there were no significant differences in the occurrence time of POD (*p* = 0.985), duration of POD (*p* = 0.355), and classification of POD (*p* = 1.000) among the R, S, and RS groups (*p* > 0.05, Table 2).

**Table 2:** Incidence and severity of postoperative delirium (POD) in the three groups.

Group	R (*n* = 30)	S (*n* = 30)	RS (*n* = 30)	*χ^2^*/*H*	*P*
Incidence of POD [*n* (%)]	4.0 (13.3%)	6.0 (20.0%)	3.0 (10.0%)	1.230	0.654
Occurrence time of POD (d)	1.0 (1.5)	1.0 (1.25)	1.0 (0)	0.031	0.985
Duration of POD (h)	12.0 (26.5)	18.5 (31.5)	17.0 (0)	2.072	0.355
Classification of POD				2.404	1.000
High active delirium [*n* (%)]	1 (25.0%)	1 (16.7%)	0 (0)		
Low activity delirium [*n* (%)]	3 (75.0%)	4 (66.7%)	2 (66.7%)		
Mixed delirium [*n* (%)]	0 (0)	1 (16.7%)	1 (16.7%)		

Occurrence time of POD is measured in days, and refers to the day on which POD occurs. R: After 0.5 μg/kg of sufentanil, the patients were first induced with remimazolam at 6 mg/kg/h until consciousness disappeared, and then maintained under anesthesia at a dose of 0.5–2 mg/kg/h. S: The patients were first given 0.5 μg/kg of sufentanil, as well as received 8% sevoflurane (oxygen flow 8 L/min), and were maintained under anesthesia with 1–2% sevoflurane after consciousness disappeared. RS: After being treated with 0.5 μg/kg of sufentanil, the patients were induced with 6 mg/kg/h remimazolam while inhaling 8% sevoflurane, and after consciousness disappeared, the patients were kept under anesthesia with 0.5–2 mg/kg/h remimazolam combined with 0.4–2% sevoflurane.

### Incidence of delayed recovery of postoperative cognitive function

Further, the incidence of delayed recovery of postoperative cognitive function was assessed. The overall rate of incidence of delayed recovery of postoperative cognitive function was 40.0% (36/90). Among them, 14 patients (46.7%) in the R group, 10 patients (33.3%) in the S group, and 12 patients (40.0%) in the RS group showed delayed recovery of postoperative cognitive function, as well as there was no evident difference in the incidence of delayed recovery of postoperative cognitive function among the R, S, and RS groups (*p* = 0.616 > 0.05, Table 3).

**Table 3:** Incidence of delayed recovery of postoperative cognitive function.

Group	R (*n* = 30)	S (*n* = 30)	RS (*n* = 30)	*χ^2^*	*p*
Incidence [*n* (%)]	14 (46.7%)	10 (33.3%)	12 (40.0%)	1.111	0.616

R: After 0.5 μg/kg of sufentanil, the patients were first induced with remimazolam at 6 mg/kg/h until consciousness disappeared, and then maintained under anesthesia at a dose of 0.5–2 mg/kg/h. S: The patients were first given 0.5 μg/kg of sufentanil, as well as received 8% sevoflurane (oxygen flow 8 L/min), and were maintained under anesthesia with 1–2% sevoflurane after consciousness disappeared. RS: After being treated with 0.5 μg/kg of sufentanil, the patients were induced with 6 mg/kg/h remimazolam while inhaling 8% sevoflurane, and after consciousness disappeared, the patients were kept under anesthesia with 0.5–2 mg/kg/h remimazolam combined with 0.4–2% sevoflurane.

### Comparison of postoperative pain score and recovery quality score among the three groups

As shown in Table 4, whether in a resting state or in a rolling over state, there were no significant differences in the NRS scores and recovery quality scores at postoperative day 1, day 2 and day 3 among the R, S and RS groups (*p* > 0.05), which indicated that these three anesthesia methods did not markedly influence the pain of patients and quality of recovery.

**Table 4:** Comparison of postoperative pain score and recovery quality score among the three groups.

Group	R (*n* = 30)	S (*n* = 30)	RS (*n* = 30)	*χ^2^*/*F*	*p*
Postoperative NRS score					
Postoperative day 1 (resting)	2.5 ± 0.8	2.1 ± 0.7	2.3 ± 0.5	1.766	0.177
Postoperative day 1 (roll over)	4.7 ± 1.1	4.2 ± 1.2	4.4 ± 0.8	1.767	0.177
Postoperative day 2 (resting)	1.5 ± 0.7	1.2 ± 0.6	1.45 ± 0.5	1.559	0.216
Postoperative day 2 (roll over)	3.4 ± 1.0	2.9 ± 0.9	3.2 ± 0.7	2.250	0.112
Postoperative day 3 (resting)	0.9 ± 0.5	0.7 ± 0.5	0.7 ± 0.5	1.887	0.158
Postoperative day 3 (roll over)	2.0 ± 0.9	2.0 ± 0.9	2.1 ± 0.5	0.128	0.880
Postoperative recovery quality score (QoR-15)					
Postoperative day 1	84.3 ± 13.3	87.2 ± 10.2	88.3 ± 9.2	1.067	0.348
Postoperative day 2	95.5 ± 10.9	9,753 ± 9.9	97.9 ± 7.2	0.557	0.575
Postoperative day 3	106.1 ± 8.7	105.2 ± 8.4	108.1 ± 10.2	0.786	0.459

NRS, Numeric Rating Scale. R: After 0.5 μg/kg of sufentanil, the patients were first induced with remimazolam at 6 mg/kg/h until consciousness disappeared, and then maintained under anesthesia at a dose of 0.5–2 mg/kg/h. S: The patients were first given 0.5 μg/kg of sufentanil, as well as received 8% sevoflurane (oxygen flow 8 L/min), and were maintained under anesthesia with 1–2% sevoflurane after consciousness disappeared. RS: After being treated with 0.5 μg/kg of sufentanil, the patients were induced with 6 mg/kg/h remimazolam while inhaling 8% sevoflurane, and after consciousness disappeared, the patients were kept under anesthesia with 0.5–2 mg/kg/h remimazolam combined with 0.4–2% sevoflurane.

### Comparison of intraoperative drug dosage, consciousness disappearance time, extubation time, and recovery time among the three groups

The induced dosages of remimazolam in the R and RS groups were 0.23 ± 0.06 and 0.17 ± 0.05 mg/kg, respectively, which showed that the induced dosage of remimazolam in the RS group was significantly lower than that in the R group (*p* = 0.002, 95% confidence interval [CI]: 0.03–0.09, Table 5). Additionally, the maintenance doses of remimazolam were, respectively, 0.79 ± 0.20 and 0.54 ± 0.11 mg/kg/h in the R and RS groups, which suggested that in comparison with the R group, the maintenance dose of remimazolam was evidently lower in the RS group (*p* < 0.001, 95% CI: 0.17–0.32, Table 5). There were no significant differences in the remifentanil dosages (*p* = 0.641) and sufentanil dosages (*p* = 0.327) among the R, S, and RS groups (*p* > 0.05, Table 5). Furthermore, the time of consciousness loss in the R, S, and RS groups were 1.56 ± 0.40, 1.24 ± 0.38, and 1.00 ± 0.10 min, respectively, as well as there was statistical difference in the time of consciousness loss among the R, S, and RS groups (*p* < 0.001) with the lowest in the RS group (Table 5). However, no significant differences were found in walking time or extubating time among the three groups (*p* > 0.05, Table 5).

**Table 5:** Comparison of intraoperative drug dosage, consciousness disappearance time, extubation time and recovery time among the three groups.

Group	R (*n* = 30)	S (*n* = 30)	RS (*n* = 30)	*χ^2^*/*t*/*F*	*p*
Induced dosage of remimazolam (mg/kg)	0.23 ± 0.06	/	0.17 ± 0.05	3.409	0.002
Maintenance dose of remimazolam (mg/kg/h)	0.79 ± 0.20	/	0.54 ± 0.11	4.988	< 0.001
Remifentanil dosage (mg)	2.6 ± 0.7	2.7 ± 0.9	2.4 ± 0.7	0.448	0.641
Sufentanil dosage (μg)	35.1 ± 6.0	33.7 ± 5.3	36.5 ± 6.8	1.139	0.327
Time of consciousness loss (min)	1.56 ± 0.40	1.24 ± 0.38	1.00 ± 0.10	14.941	< 0.001
Waking time (min)	15.80 ± 7.70	13.70 ± 5.62	18.10 ± 10.30	1.481	0.236
Extubation time (min)	18.70 ± 7.90	16.00 ± 6.84	20.95 ± 9.94	1.771	0.179

R: After 0.5 μg/kg of sufentanil, the patients were first induced with remimazolam at 6 mg/kg/h until consciousness disappeared, and then maintained under anesthesia at a dose of 0.5 to 2 mg/kg/h. S: The patients were first given 0.5 μg/kg of sufentanil, as well as received 8% sevoflurane (oxygen flow 8 L/min), and were maintained under anesthesia with 1–2% sevoflurane after consciousness disappeared. RS: After being treated with 0.5 μg/kg of sufentanil, the patients were induced with 6 mg/kg/h remimazolam while inhaling 8% sevoflurane, and after consciousness disappeared, the patients were kept under anesthesia with 0.5–2 mg/kg/h remimazolam combined with 0.4–2% sevoflurane.

### Comparison of the adverse reactions among the three groups

Finally, the occurrence of adverse reactions among patients in the three groups was compared. There were 4, 2, and 3 patients, respectively, in the R, S, and RS groups with waking agitation, with no significant difference (*p* = 0.905 > 0.05; Table 6). No patients in the R, S, or RS groups experienced intraoperative awareness and intraoperative movement (Table 6). The incidences of intraoperative hypotension in the R, S, and RS groups were 23.37, 56.7, and 43.3%, respectively, and differed statistically among the three groups (*p* = 0.031 < 0.05; Table 6). Compared with the R group, the incidence of intraoperative hypotension was significantly higher in the S group (*p* = 0.008, 95%CI: 1.4–13.1), while it was not evidently changed in the RS group (*p* = 0.100, 95%CI: 0.8–7.6; Table 6). Moreover, no significant differences were observed in the incidences of intraoperative tachycardia and bradycardia among the R, S, and RS groups (*p* > 0.05, Table 6).

**Table 6:** Comparison of the adverse reactions among the three groups.

Group	R (*n* = 30)	S (*n* = 30)	RS (*n* = 30)	*χ* ^2^	*p*
Waking agitation [*n* (%)]	4 (13.3%)	2 (6.7%)	3 (10%)	0.797	0.905
Intraoperative awareness [*n* (%)]	0 (0%)	0 (0%)	0 (0%)		
Intraoperative movement [*n* (%)]	0 (0%)	0 (0%)	0 (0%)		
Hypotension [*n* (%)]	7 (23.37%)	17 (56.7%)	13 (43.3%)	6.976	0.031
Hypertension [*n* (%)]	5 (16.7%)	2 (6.7%)	3 (10.0%)	1.498	0.592
Tachycardia [*n* (%)]	3 (10.0%)	1 (3.3%)	2 (6.7%)	1.102	0.868
Bradycardia [*n* (%)]	2 (6.7%)	6 (20.0%)	3 (10.0%)	2.466	0.366

R: After 0.5 μg/kg of sufentanil, the patients were first induced with remimazolam at 6 mg/kg/h until consciousness disappeared, and then maintained under anesthesia at a dose of 0.5–2 mg/kg/h. S: The patients were first given 0.5 μg/kg of sufentanil, as well as received 8% sevoflurane (oxygen flow 8 L/min), and were maintained under anesthesia with 1–2% sevoflurane after consciousness disappeared. RS: After being treated with 0.5 μg/kg of sufentanil, the patients were induced with 6 mg/kg/h remimazolam while inhaling 8% sevoflurane, and after consciousness disappeared, the patients were kept under anesthesia with 0.5–2 mg/kg/h remimazolam combined with 0.4–2% sevoflurane.

## Discussion

POD is a prevalent complication following surgery and anesthesia in elderly patients, which can elevate the incidence of postoperative complications, lead to permanent cognitive function impairment, seriously impact the prognosis, and thus augment both personal and social economic burdens (43). The relationship between general anesthesia medications and changes in postoperative cognitive function has attracted attention. There has been an ongoing debate regarding the pros and cons of commonly used inhaled anesthetics (such as sevoflurane) and intravenous anesthetics (like remimazolam) in general anesthesia with respect to POD and cognitive function (44). In this prospective randomized controlled trial, our results showed that, compared with sevoflurane, remimazolam did not increase the incidence of POD or delay the recovery of early cognitive function in elderly patients undergoing laparoscopic-assisted gastrointestinal surgery, and was associated with a lower incidence of intraoperative hypotension. When combined with sevoflurane, the induction and maintenance doses of remimazolam were reduced, and the time of consciousness loss was shortened, whereas there were no significant differences in postoperative recovery time and quality.

Previous studies have proposed that the use of benzodiazepines may exacerbate and prolong POD and cognitive dysfunction in patients (24, 45). However, a recent study by Wang et al. (46) found no association between preoperative midazolam administration and delirium on the first postoperative day (*p* = 0.67, 95% CI: 0.65–1.29). Flumazenil is a specific antagonist of benzodiazepines (47). In clinical practice, it is not administered routinely; instead, it is used only when patients experience delayed awakening or poor recovery quality after benzodiazepine use (48). In the present study, flumazenil was not used during the awakening period. This decision was based on the fact that our observational indicators included awakening time and emergence agitation, parameters that would be interfered with by the administration of an antagonist. A protocol was predefined that flumazenil would be considered for administration if any enrolled patient developed delayed awakening (defined as awakening time > 60 min). However, no such cases of delayed awakening were observed throughout the trial.

Remimazolam and midazolam both belong to the benzodiazepine class, sharing similar mechanisms of action. A study of remimazolam sedation in healthy adults found faster psychomotor recovery yet mild and transient residual effects on cognitive function (49). Another study compared the effects of remimazolam and dexmedetomidine on postoperative early cognitive dysfunction in elderly patients with gastric cancer. It illustrated that both remimazolam and dexmedetomidine were beneficial in reducing the incidence of early POD in elderly patients after radical gastric cancer resection, but the incidence of adverse reactions (such as hypotension, bradycardia, and respiratory depression) after remimazolam treatment was lower (50). Additionally, a recent randomized controlled trial showed that the intraoperative administration of remimazolam did not increase the incidence of PODs in elderly patients undergoing orthopedic surgery but did reduce the incidence of post-induction hypotension and decreased the intraoperative dose of vasoactive drugs (51). In the current study, we observed that, compared with sevoflurane, remimazolam did not increase the incidence of POD or delay the recovery of postoperative cognitive function in elderly patients, which may be related to its unique metabolic characteristics. As a new ultra-short-acting drug, it may reduce the adverse effects of anesthetic drug accumulation on patients (52).

Regarding the commonly used inhaled and intravenous anesthetics in clinical practice, there are differing views on their impacts on POD and cognitive function. A meta-analysis showed no significant difference in the incidence of POD after non-cardiac surgery between intravenous anesthesia and inhalation anesthesia (*p* > 0.05, odds ratio 0.59, 95% CI: 0.15–2.26) (10). In a randomized controlled study, 544 elderly patients undergoing laparoscopic abdominal surgery were randomly divided into two groups, and no significant difference was found in the incidence of delayed neurocognitive recovery after intravenous (propofol) and inhalation (sevoflurane) anesthesia (53). In our experiment, we likewise observed no significant differences in the effects of remimazolam, sevoflurane, or their combination on POD and delayed recovery of postoperative cognitive function in the older adult patients undergoing laparoscopic gastrointestinal surgery.

Furthermore, our trial found that the combination of remimazolam and sevoflurane could reduce the induction and maintenance doses of remimazolam and shorten the duration of loss of consciousness. Huang et al. demonstrated that remimazolam could effectively lower the minimum alveolar concentration. of sevoflurane at the end-expiration period during laryngeal mask placement in adults, indicating a synergistic effect between remimazolam and sevoflurane. Moreover, when remimazolam and sevoflurane are used in combination, the dose of remimazolam may be reduced (37). A previous study reported that midazolam combined with sevoflurane for general anesthesia could prolong patients’ waking and recovery times (54). However, this study showed that the combination of remimazolam and sevoflurane did not extend the patients’ awake time and extubation time. This may be attributed to the unique metabolic mode of remimazolam, which is metabolized by non-specific esterase hydrolysis, independent of liver and kidney function, and the metabolites are inactive and difficult to accumulate (55). In this experiment, no intraoperative awareness or movement occurred in any of the three groups. Furthermore, there were no significant differences in the incidence of adverse reactions, including hypertension, tachycardia, and bradycardia, among the three groups. In comparison with the S group, the incidence of intraoperative hypotension and the amount of norepinephrine used in the R group were evidently lower. These results suggest that hemodynamics after remimazolam induction are more stable, consistent with previous research results (56, 57). Hypotension is often associated with adverse outcomes, especially in older adult patients with more comorbidities, and may contribute to a decline in perioperative cognitive function (58). The stable hemodynamic properties of remimazolam may be closely correlated with the non-increased incidence of POD.

The differential impacts of sevoflurane and remimazolam on cognitive function, as well as the synergistic effects of their combination, are mediated by divergent yet complementary mechanisms. Sevoflurane impairs cognition mainly via neuroinflammation and synaptic dysfunction. In animal models, it activates central nervous system-resident microglia, prompting the release of pro-inflammatory cytokines (e.g., TNF-*α*, IL-6) that cross the blood–brain barrier, thereby reducing synaptic density and disrupting glutamate-NMDA receptor signaling, critical for learning and memory (59, 60). Additionally, sevoflurane modulates *γ*-aminobutyric acid (GABA) receptors; prolonged GABA activation hyperpolarizes neurons, suppresses hippocampal neurogenesis, and alters memory-relevant oscillatory brain activity (61). Notably, the dose dependence of sevoflurane’s cognitive effects (62) may explain why the sevoflurane treatment showed no significant increase in POD vs. remimazolam, as limited exposure mitigates these adverse pathways. Conversely, remimazolam’s cognitive safety derives from unique pharmacokinetic and pharmacodynamic traits: unlike midazolam, it has a shorter receptor residence time, reducing prolonged GABAergic suppression of hippocampal activity (19, 63). Moreover, its minimal hemodynamic effects indirectly protect cognition by maintaining stable blood pressure and preventing cerebral hypoperfusion/neuronal hypoxia (64), while preclinical studies confirm it does not trigger hippocampal cell death (unlike high-dose propofol) (65, 66). For the combination of sevoflurane and remimazolam, synergy arises from complementary actions: sevoflurane’s rapid GABA activation accelerates induction, while remimazolam’s lower GABA receptor affinity reduces total anesthetic load, synergistically lowering remimazolam dosage to minimize residual cognitive effects, and reducing sevoflurane concentration to blunt microglial activation/cytokine release (67–69). Remimazolam’s hemodynamic stability also offsets sevoflurane’s nitric oxide-mediated vasodilation, explaining the RS group’s lower hypotension rate vs. the S group.

Notably, the contribution of anesthetic agents to POD development remains a topic of ongoing controversy in the field. While some studies have suggested that specific anesthetics (e.g., sevoflurane) may be associated with a higher risk of POD due to neurotoxic effects (70, 71), others, including comparative studies of regional versus general anesthesia, have failed to demonstrate meaningful differences in POD incidence. This inconsistency highlights that POD is a complex, multifactorial condition, with patient-related factors (e.g., age, comorbidities, frailty), surgical factors (e.g., duration, invasiveness), and perioperative management (e.g., analgesia, fluid balance) playing equally critical roles alongside anesthetic choice (72, 73). In controlled experimental settings, like the present study, different anesthetic regimens often exhibit comparable POD propensities, as the influence of anesthetic agents may be attenuated by standardized perioperative care and strict inclusion/exclusion criteria (50, 59). Our results align with this observation: despite the prior hypothesis that sevoflurane and remimazolam might differ in POD risk, we found no significant differences in POD incidence, duration, or severity across the three groups. It is important to acknowledge that the small sample size (30 patients per group) may have limited our ability to detect potential small but clinically meaningful differences between the anesthetic regimens. While our sample size was calculated based on prior literature reporting large effect sizes (6.9% vs. 26.7% POD incidence between intravenous and inhalational anesthesia (41)), it may lack sufficient power to identify subtle differences (e.g., <10% absolute difference in POD rates) that could be clinically relevant in large cohorts. This limitation should be considered when interpreting the null findings regarding POD, as the comparable rates do not necessarily rule out modest but real effects of anesthetic choice on POD development.

However, this trial has some limitations. First, its single-center, small-sample design (90 patients from one tertiary hospital) and strict exclusion criteria (e.g., excluding those with severe hepatic/renal dysfunction or pre-existing cognitive impairment) limit generalizability to community hospitals or patients with more comorbidities, and may have bias. Additionally, another potential limitation of this study is the selection of anesthesia induction regimens, which may also restrict the generalizability of our findings regarding the synergistic effect of remimazolam combined with sevoflurane on shortening induction time. Second, cognitive function was only assessed up to postoperative day 3, missing long-term outcomes (e.g., 3–6 months of cognitive decline); given POD’s association with persistent cognitive impairment, delayed anesthetic effects on cognition cannot be ruled out. Third, unmeasured perioperative biomarkers (e.g., IL-6, TNF-*α*, BDNF) prevented validation of proposed mechanisms (e.g., sevoflurane-induced inflammation). Finally, the absence of subgroup analyses by age (≥75 vs. 65–74 years) or frailty stage (known POD risk factors) means remimazolam’s potential greater benefits in the oldest-old or frail patients remain unclear. To address these limitations, future research should: (1) conduct multi-center trials with ≥500 patients (across tertiary/community hospitals), stratified by age, frailty, and surgical duration, to clarify remimazolam/RS combination benefits in specific subgroups; (2) extend cognitive follow-up to 6–12 months using tools like the Montreal Cognitive Assessment (MoCA), especially for patients with pre-existing mild cognitive impairment; (3) validate mechanisms via biomarkers (cytokines), and MRI (hippocampal volume); (4) compare remimazolam with other regimens (e.g., dexmedetomidine, propofol-sevoflurane) to identify optimal strategies for high-risk elderly patients; (5) develop individualized anesthesia via pharmacogenomic testing (e.g., esterase gene variants for remimazolam metabolism) to advance geriatric precision anesthesia.

In conclusion, our study showed that in elderly patients undergoing laparoscopic-assisted gastrointestinal surgery, general anesthesia with remimazolam was not associated with an increased incidence of POD or delayed recovery of early cognitive function compared to sevoflurane. It also significantly reduced the incidence of intraoperative hypotension and the use of norepinephrine. Additionally, the combination of remimazolam and sevoflurane could reduce the induction and maintenance doses of remimazolam and shorten the time to loss of consciousness. However, larger-scale research is needed to validate these conclusions. Our trial is a preliminary study that raises new considerations regarding the use of anesthesia approaches traditionally thought to carry higher risks in individuals susceptible to POD.

Our findings support the safety of remimazolam in elderly patients at risk for POD. The lower incidence of hypotension and reduced vasopressor requirement suggest that remimazolam may be preferable in patients with cardiovascular vulnerability. Furthermore, the combination of remimazolam with sevoflurane allowed for lower doses of both agents, potentially minimizing dose-related side effects without compromising recovery quality. These insights may guide anesthesiologists in tailoring anesthesia regimens for high-risk geriatric populations.

## Data Availability

The original contributions presented in the study are included in the article/Supplementary material; further inquiries can be directed to the corresponding author.
