# Supplementary material for: Effects of remimazolam and sevoflurane on postoperative delirium and early cognitive impairment in elderly patients after laparoscopic-assisted gastrointestinal surgery: a randomized clinical trial
Source: Front Med (Lausanne). 2026 Mar 20;13:1680473. doi: 10.3389/fmed.2026.1680473 (PMC13047138; doi:10.3389/fmed.2026.1680473)
Supplement: Supplementary file 1 [file Data_Sheet_1.docx]

1. ***Measurement of observational leading indicators***

The 3-minute Diagnostic Interview for Confusion Assessment Method (3D-CAM) and the Richmond Sedation Scale (RASS) were used for POD assessment. All researchers received comprehensive training in accordance with the Chinese version of the 3D-CAM scale (**Table S1**) and the RASS (**Table S2**). The training of evaluators is supervised by the corresponding author, and all evaluators must obtain approval before starting the evaluation. The evaluation is supervised by a professional neurologist. Once a week, researchers submit any disputed cases to a third evaluator or the corresponding author. According to the RASS, the level of consciousness assessed before assessing delirium is used to evaluate the depth of sedation in patients. If the score is -4 or -5, delirium assessment is stopped. If the score is ≥ -3, delirium assessment is continued. POD patients are further divided into three subtypes: high activity delirium (+1 to+4) is defined when RASS remains positive; when RASS remains neutral or negative (-3 to 0), low activity delirium is defined; when high activity and low activity delirium occur simultaneously during the observation period, it is defined as mixed delirium, and the onset and duration of delirium are recorded. Once postoperative delirium is diagnosed, record the onset time of the delirium, conduct follow-ups every 3 hours, determine its duration, and classify the type of delirium based on the RASS scale score.

**Table S1** The 3D-CAM scale (in English)

| Explanation: Incorrect includes “don't know” and “no response/meaningless response”. For any “incorrect” or “correct” response, check the checkbox in the last column for the corresponding feature. | | | CAM features | | | |
| --- | --- | --- | --- | --- | --- | --- |
| **Introduction: I want to ask you some questions about thinking and memory** | | | 1 | 2 | 3 | 4 |
| 1 | May I ask which year it is this year? | □incorrect  □correct |  |  |  |  |
| 2 | May I ask what day it is today? | □incorrect  □correct |  |  |  |  |
| 3 | Excuse me, what place is this? (Answering "hospital" is correct) | □incorrect  □correct |  |  |  |  |
| 4 | Read some numbers. Please repeat one of them in the reverse order of the one I read. For example, if I say “5 – 2”, you should say “2 – 5”. Are you clear? The first group of numbers is “7 - 5 – 1” (1 - 5 - 7). | □incorrect  □correct |  |  |  |  |
| 5 | The second group of numbers is “8-2-4-3” (3-4-2-8). | □incorrect  □correct |  |  |  |  |
| 6 | Could you start counting down from Sunday? (Sunday, Saturday, Friday, Thursday, Wednesday, Tuesday, Monday), and you can give a hint at most twice, for example: what day was the day before Monday? | □incorrect  □correct |  |  |  |  |
| 7 | Can you count backward from December? (December, November, October, September, August, July, June, May, April, March, February, January. You can give a hint up to 2 times: what was the month before March?) | □incorrect  □correct |  |  |  |  |
| 8 | Have you felt confused on this recent day? | □Yes  □No |  |  |  |  |
| 9 | (If the answer to Question 3 is "Incorrect", just mark "Yes" without further questioning. Otherwise, you need to be asked:) Did you feel that you weren't in the hospital on this recent day? | □Yes  □No |  |  |  |  |
| 10 | Have you witnessed anything that doesn't actually exist recently? | □Yes  □No |  |  |  |  |
| **Observer evaluation: Ask the patient questions 1-10 above to complete** | | | | | | |
| 11 | During the assessment, did the patient show signs of drowsiness, stupor or coma? | □Yes  □No |  |  |  |  |
| 12 | Does the patient exhibit excessive sensitivity and excitement towards the routine things in the environment (increased alertness)? | □Yes  □No |  |  |  |  |
| 13 | Does the patient have difficulty in thinking clearly or logically, such as recounting irrelevant matters (deviating from the topic) during the conversation? | □Yes  □No |  |  |  |  |
| 14 | Does the patient talk in an aimless manner, such as having inappropriate rambling or answering irrelevant questions? | □Yes  □No |  |  |  |  |
| 15 | Has the patient's language significantly decreased compared to usual? (For example, answer with “Yes” or “No”) | □Yes  □No |  |  |  |  |
| 16 | During the assessment, did the patient fail to keep up with the topic being discussed? | □Yes  □No |  |  |  |  |
| 17 | Did the patient exhibit inappropriate distraction due to environmental stimuli? | □Yes  □No |  |  |  |  |
| 18 | During the assessment, did the patient have fluctuations in consciousness level? For instance, responding appropriately at the beginning and then falling into a confused sleep state. | □Yes  □No |  |  |  |  |
| 19 | During the assessment, did the patient have fluctuations in attention level? For example, there were significant changes in the patient's focus on the conversation or performance on attention tests? | □Yes  □No |  |  |  |  |
| 20 | During the assessment process, were there any changes in the patient’s language expression or thinking? For instance, did the patient's speaking speed vary from fast to slow? | □Yes  □No |  |  |  |  |
| **Optional question: If only Feature 1 is not selected, while Feature 2 and Feature 3 or Feature 4 are selected, proceed.** | | | | | | |
| 21 | Ask the family members, friends or medical staff who have a very good understanding of the patient's condition: “Are there any signs indicating that the patient has experienced an acute change in mental state (memory or thinking) compared to their usual condition?” | □Yes  □No |  |  |  |  |
| 22 | If the results of this hospitalization or previous 3D-CAM evaluations are available, please compare them. Based on the newly emerged "positive" items this time, determine whether the patient has experienced any acute changes. | □Yes  □No |  |  |  |  |
| Conclusion | Did the above list include all four characteristics of CAM? | CAM | 1 | 2 | 3 | 4 |
|  | Check whether the corresponding CAM features are present in the above list. Please follow the diagnostic criteria: Feature 1 + Feature 2 + Feature 3 or 4. | □delirium  □no delirium | | | | |

Note: In the 3D-CAM assessment, if a patient perceives something that does not actually exist, they will consider it as postoperative delirium!

**Table S2** The RASS scale (in English)

| +4 | Aggressive | Aggressiveness or violence, poses a direct danger to the staff. |
| --- | --- | --- |
| +3 | Very restless | Try to remove the breathing tube, gastric tube or intravenous drip, and exhibit aggressive behavior towards the staff. |
| +2 | Restlessness | The patient’s body moved vigorously, and their breathing was out of sync with that of the ventilator. |
| +1 | Anxiety and unease | Anxious and tense, but with only slight body movements and no aggression. |
| 0 | Alert and calm | Conscious natural state. |
| -1 | Drowsy | Not fully awake, but remained conscious to auditory stimuli and maintained eye contact for more than 10 seconds. |
| -2 | Mild sedation | Remaining alert to auditory stimuli and maintaining eye contact for less than 10 seconds. |
| -3 | Moderate sedation | Responds to auditory stimuli, but has no eye contact. |
| -4 | Deep sedation | No response to auditory stimuli, but responsive to physical stimuli. |
| -5 | Coma | There is no response to either auditory stimuli or physical stimuli. |

1. ***Measurement of the incidence of delayed recovery of postoperative cognitive function using MMSE***

The specific content of the MMSE for adults is shown in **Table S3**. Scores were recorded on the preoperative day and the postoperative days 1, 2, and 3. If the MMSE score on the preoperative day was less than 17 for illiteracy, less than 20 for primary school education, and less than 24 for secondary school education (including vocational school), cognitive impairment was identified and excluded. No cognitive impairment was included in the trial. Assess whether there is early postoperative cognitive impairment: MMSE score for illiteracy <17 points, primary school level <20 points, secondary school level (including technical secondary school) <24 points, or a score 2 points lower than preoperative level, indicating cognitive impairment. If the patient experiences postoperative delirium, the assessment will be postponed until postoperative delirium is restored.

**Table S3** The MMSE scale

| Please circle the score. | Integral | | | | | | |
| --- | --- | --- | --- | --- | --- | --- | --- |
| 1. Which year is it this year?  2. What season is it now?  3. What month is it now?  4. What is today's date?  5. What day of the week is it today? |  |  |  |  | 1  1  1  1  1 |  | 0  0  0  0  0 |
| 6. Where are we now?  7. Where are we now in the district?  8. Where are we now on the street?  9. Where is the current hospital?  10. Which floor is this? |  |  |  |  | 1  1  1  1  1 |  | 0  0  0  0  0 |
| 11. Tell you three things. After I finish speaking, please repeat them. Tree, clock, car (1 point each, total 3 points) | 3 |  | 2 |  | 1 |  | 0 |
| 12. 100－7=?Five consecutive times (1 point each, totaling 5 points) | 5 | 4 | 3 | 2 | 1 |  | 0 |
| 13. Now please tell me what I asked you to remember just now (1 point each, for a total of 3 points) |  |  | 3 | 2 | 1 |  | 0 |
| 14. (Show the watch) What's this called?  (Show the pen) What's this called? |  |  |  |  | 1 |  | 0 |
| 15. Please tell me “Good harvest follows a snowy winter.” |  |  |  |  | 1 |  | 0 |
| 16. I’ll give you a piece of paper. Please follow what I say and do it now. Start: “Hold this paper with your right hand, then fold it in half with both hands and place it on your left leg.” (1 point for each item, total 3 points). | 3 |  | 2 |  | 1 |  | 0 |
| 17. Please repeat this sentence and do as instructed: “Close your eyes.” |  |  |  |  | 2 |  | 0 |
| 18. Please write me a complete sentence (no names please). |  | | | | 1 |  | 0 |
| 19. (Presenting the pattern) Please draw it exactly as shown here. |  | | | | 1 |  | 0 |

**Rating criteria:**

A score of 27-30: A normal score of less than 27 may indicate cognitive impairment; Dementia classification criteria: Illiteracy <17 points, primary school level <20 points, secondary school level (including vocational school) <24 points

*Assess MMSE the day before surgery, and exclude cognitive impairment if present.

1. ***Adverse event definitions***

All intraoperative and postoperative adverse events were defined in accordance with standard clinical criteria and previously published anesthesia research:

(1) Intraoperative hypertension: A transient or sustained increase in systolic blood pressure (SBP) ≥ 20% above the patient’s baseline SBP (measured after entering the operating room and before anesthesia induction) or SBP > 160 mmHg, lasting for ≥ 1 minute.

(2) Intraoperative hypotension: A transient or sustained decrease in SBP ≥ 20% below the patient’s baseline SBP or SBP < 90 mmHg, lasting for ≥ 1 minute.

(3) Intraoperative tachycardia: A transient or sustained increase in heart rate (HR) ≥ 20% above the patient’s baseline HR or HR > 100 beats per minute (bpm), lasting for ≥ 1 minute.

(4) Intraoperative bradycardia: A transient or sustained decrease in HR ≥ 20% below the patient’s baseline HR or HR < 50 bpm, lasting for ≥ 1 minute.

(5) Arrhythmia: Any deviation from the normal sinus rhythm detected by electrocardiography (ECG), including but not limited to atrial premature beats (≥ 5 beats per minute), ventricular premature beats (≥ 3 beats per minute), atrial fibrillation, or atrioventricular block (≥ second degree).

(6) Injection pain: Patient-reported discomfort, burning, or stabbing pain at the intravenous cannulation site during the administration of anesthetic drugs (e.g., remimazolam), assessed via verbal confirmation (e.g., “Do you feel pain at the needle site?”).

(7) Postoperative nausea: Subjective sensation of abdominal discomfort or the urge to vomit, reported by the patient within 24 hours after surgery.

(8) Postoperative vomiting: Forceful expulsion of gastric contents through the mouth, observed by researchers or reported by the patient within 24 hours after surgery.

(9) Postoperative dizziness: Patient-reported sensation of lightheadedness, unsteadiness, or spinning (vertigo) when transitioning from a supine to a sitting or standing position, assessed during postoperative rounds within 24 hours after surgery.
